# Low education and employment status drive cardiometabolic health: a social determinants perspective in Chinese population

**DOI:** 10.3389/fpubh.2025.1676833

**Published:** 2025-12-10

**Authors:** Jia-feng Zhang, Xin-yi Cai, Wei Tang, Yan Song, Xing-xing Zhang, Yong-quan Shi, Tuo Li

**Affiliations:** 1 Department of Laboratory Medicine, Changzheng Hospital, Shanghai, China; 2 Department of Endocrinology, Changzheng Hospital, Shanghai, China

**Keywords:** cardiovascular-kidney-metabolic (CKM) syndrome, education, employment, income, social determinants of health (SDOH)

## Abstract

**Background:**

Cardiovascular-kidney-metabolic (CKM) syndrome has emerged a major strain on healthcare systems worldwide, with marked disparities in incidence across different regions, which are partly attributed to social determinants of health (SDOH). While research has touched upon the link between SDOH and CKM, and isolated SDOH factor’s influence on cardiovascular health has been probed in China, evaluation of how SDOH collectively affect CKM in Chinese population remains underexplored in a systematic fashion.

**Methods:**

The study drew on Shanghai community population (STONE cohort, *n* = 4,066) recruited via a three-phase stratified sampling scheme. Standardized interviews captured education, marital status, employment, household income, and neighborhood characteristics as SDOH exposures. CKM stages 0–4 were assigned using American Heart Association (AHA) criteria. Multinomial logistic regression quantified SDOH–CKM relationships, with age-, sex-, and lifestyle-stratified analyses to pinpoint vulnerable subgroups.

**Results:**

Unfavorable SDOH profiles, including limited schooling (OR = 8.18; 95% CI: 6.16–10.86), unemployment or retirement (OR = 11.38; 95% CI: 8.59–15.08), and low income (OR = 2.24; 95% CI: 1.78–2.81) were robustly linked to CKM advancement independent of classic metabolic risk factors. The effect was present only among adults aged 36–50 years (OR = 2.45; 95% CI: 1.37–4.40). Women clear of alcohol displayed elevated odds with advanced CKM stages, whereas men did not show significant association with CKM progression.

**Conclusion:**

Unfavorable SDOH, especially low education and unemployed or retired, and limited income, constitute independent determinants of CKM progression. Embedding SDOH metrics into routine CKM surveillance and designing targeted interventions for mid-life adults and women could transform primary prevention strategies.

## Introduction

The prevalence of metabolic dysfunction-related diseases, chronic kidney disease (CKD) and cardiovascular diseases (CVD), have emerged as significant global public health concerns (1–3). In light of these insights, the American Heart Association (AHA) innovatively proposed the concept of Cardiovascular-Kidney-Metabolic (CKM) syndrome and further devised a stages 0–4 for the assessment of cardio-metabolic health, offering a new paradigm for integrated prevention and management (4, 5). Epidemiological data reveal significant geographic disparities in the prevalence of CKM worldwide. In United States and Korea, CKM-0 was observed in 11.2 ~ 21.1% participants, with a prevalence of CKM-3/4 reaching 10.1 ~ 13.4% (6, 7).

These disparities can be attributed to the differing distributions of traditional metabolic risk factors, and to the prominent impact of various conditions such as housing and socioeconomic status, namely, social determinants of health (SDOH) as well, comprising economic stability, neighborhood and physical environment, education, food security, community and social context, and healthcare system (8, 9). Previous studies have explored the impact of SDOH on cardiovascular health in U.S. populations (10, 11). Parallel efforts in China have delved into the ways educational attainment and socioeconomic standing relate to the components of CKM (12–14). However, there remains a notable gap in systematic research focusing on the relation of holistic SDOH and CKM staging in the context of Chinese population, taking demographic information and metabolic dysfunctions into account.

Therefore, the study explored the associations between five key SDOH indicators including education level, marital status, employment status, annual income, and community type with CKM staging, in a community-based sample in Shanghai, aiming to provide robust scientific evidence to inform the development of targeted social intervention strategies.

## Materials and methods

### Study design and population

This cross sectional study was conducted among residents of Shanghai, China, conducted from 2016 to 2018. The study protocol was approved by the Shanghai Changzheng Hospital Ethics Committee (2016SL039, NCT07043166) and adhered to the Declaration of Helsinki. All participants provided written informed consent prior to enrollment.

### Sampling strategy and participants

A three-stage stratified sampling strategy was employed to obtain a representative sample of Shanghai’s demographic distribution across 18 districts and 242 community health service centers. First, considering the demographic distribution across Shanghai, 10 community health centers were randomly selected (4 in urban areas, 3 in urban–rural areas, and 3 in rural areas). Second, residential communities provided household composition information, and random sampling was applied to select households. Finally, the KISH grid method was employed to determine individual participants for survey (15).

### Inclusion criteria

**Figure 1:**
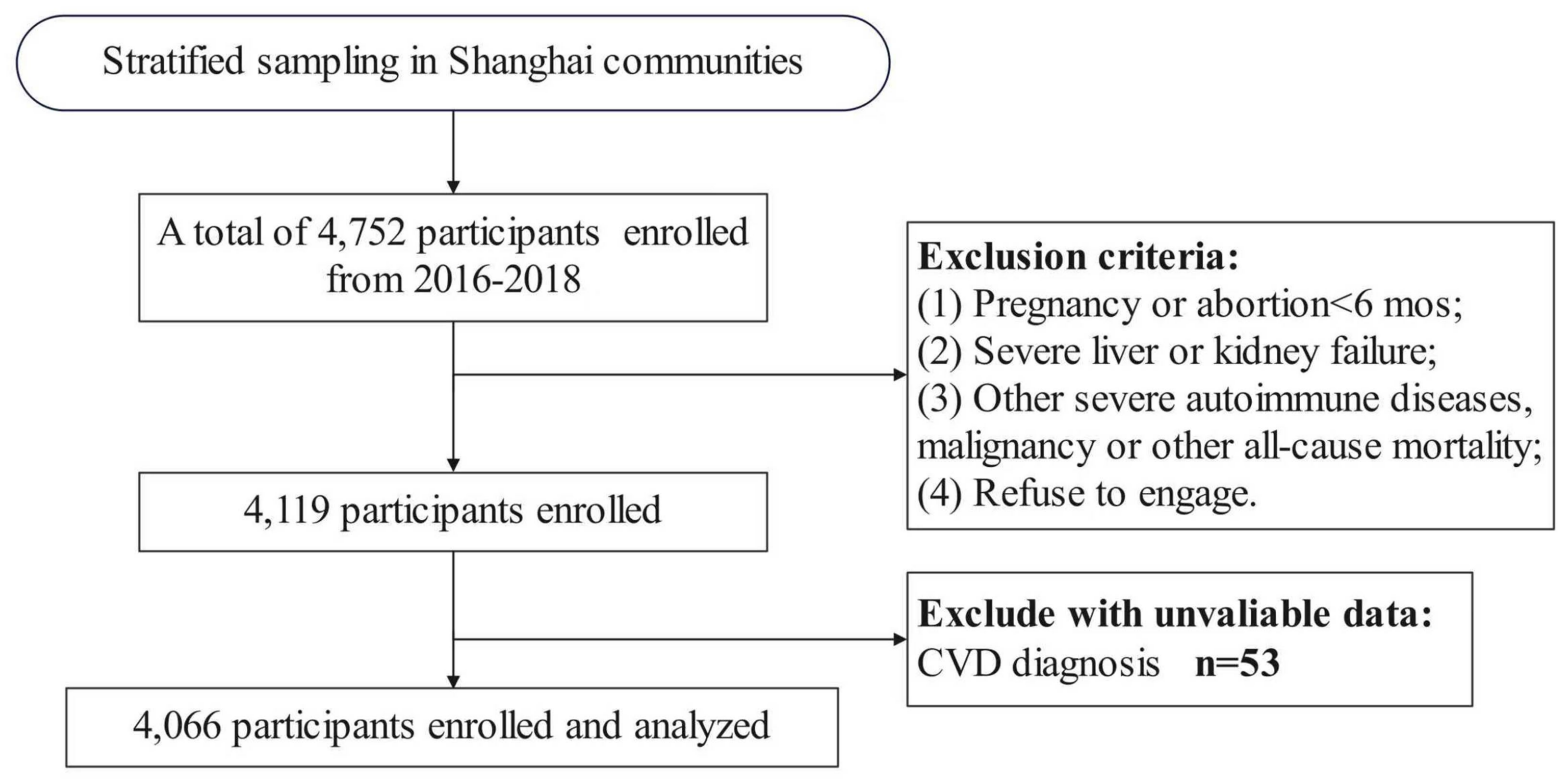
Flow chart illustrating the participant selection process, STONE population.

Adults aged 18–65 years who had resided in the selected community for more than 6 months and were free of severe diseases. Exclusion criteria: (1) pregnancy or abortion within 6 months; (2) severe liver or kidney failure; (3) severe autoimmune diseases, solid or hematologic malignancy, or terminal illness; (4) refusal to participate. A total of 4,752 residents were approached, of whom 4,119 consented to participate (participation rate: 86.7%). After applying exclusion criteria, 4,066 participants were included in the final analysis (Figure 1).

### Data collection

Comprehensive data collection was conducted at each community health center, including structured questionnaires, anthropometric measurements, laboratory assessments (blood and urine samples), ultrasonography, and dual-energy X-ray absorptiometry (DXA). Trained healthcare providers conducted confidential interviews using standardized questionnaires covering demographics, socioeconomic status, lifestyle factors, and health-related behaviors.

### Social determinants of health assessment

SDOH were assessed using five key domains based on established frameworks (10) and adapted for the Chinese population. For each domain, participants were classified into advantage (favorable) or disadvantage (unfavourable) as follows:

1. Educational attainment: High school graduate as higher education *vs.* less than high school education as lower education.
2. Marital status: married or living with a partner (coupled) *vs.* unmarried/not living with a partner (single).
3. Employment status: Employed or student *vs.* retired or unemployed.
4. Household income: Annual income ≥ 88,366 Chinese Yuan (higher) *vs.* < 88,366 Chinese Yuan (lower) (from Shanghai Municipal Bureau of Statistics).^1^
5. Community type: Urban vs. suburban or rural residence.
6. Each unfavorable SDOH domain was scored as 1 point, while favorable conditions were scored as 0 points. The total SDOH score ranged from 0 to 5, with scores ≥ 2 classified as favorable overall SDOH status and < 2 as unfavorable overall SDOH status.

### CKM syndrome staging

CKM syndrome stages were defined according to the AHA presidential advisory classification system, ranging from CKM-0 to 4 (4). CKM-0: No risk factors (BMI 18.5–24.9 kg/m^2^, waist circumference < 85/90 cm for women/men, no metabolic disorders); CKM-1: Excess/dysfunctional adipose tissue (BMI ≥ 25 kg/m^2^ or central obesity with waist circumference ≥85/90 cm for women/men, or prediabetes); CKM-2: Metabolic risk factors (diabetes, dyslipidemia, hypertension, or moderate CKD); CKM-3: Subclinical CVD or high-risk CKD; CKM-4: Clinical CVD (coronary heart disease, stroke, heart failure, or peripheral artery disease). Advanced CKM stages were defined as CKM-3/4, while non-advanced stages included stages 0, 1, or 2.

### Clinical definitions

Obesity: BMI ≥ 28 kg/m^2^ and/or waist circumference ≥ 85/90 cm for women/men according to Chinese guidelines (16). Diabetes: Fasting plasma glucose ≥ 7.0 mmol/L, 2-h post-load glucose ≥ 11.1 mmol/L, HbA_1c_ ≥ 6.5%, or use of diabetes medications (17). Dyslipidemia: Total cholesterol ≥ 6.2 mmol/L, triglycerides ≥ 2.3 mmol/L, low-density lipoprotein-cholesterol (LDL-C) ≥ 4.1 mmol/L, or high-density lipoprotein-cholesterol (HDL-C) < 1.0 mmol/L (18). Hypertension: Systolic blood pressure ≥ 140 mmHg, diastolic blood pressure ≥ 90 mmHg, or use of antihypertensive medications (19). CKD: Estimated glomerular filtration rate < 60 mL/min/1.73m^2^ or albumin-to-creatinine ratio (ACR) ≥ 30 mg/g. Moderate risk: eGFR 45–59 mL/min/1.73 m2 with ACR < 30 mg/g, or eGFR≥60 mL/min/1.73 m2 with ACR 30–299 mg/g. High risk: eGFR 30–44 mL/min/1.73 m2 with ACR < 30 mg/g, or eGFR 45–59 mL/min/1.73 m2 with ACR 30–299 mg/g, or eGFR≥60 mL/min/1.73 m2 with ACR ≥ 300 mg/g (20). CVD: Presence or self-reported history of coronary heart disease, stroke, or heart failure.

### Statistical analysis and visualization

Statistical analyses were performed using *R* software (*version 4.3.0*). Continuous variables were presented as mean ± standard deviation and compared using ANOVA. Categorical variables were presented as frequencies and percentages, with differences assessed using chi-square tests.

Among the 4,066 participants, 408 individuals (10.0%) had at least one missing variable. Simple imputation (mean for continuous variables and mode for categorical variables) was applied to these participants before analysis. Multinomial logistic regression models were used to examine associations between SDOH and CKM stages, with odds ratios and 95% confidence intervals reported. Three sequential models were constructed with progressive adjustment for confounders: Model 1 adjusted for sex only; Model 2 additionally adjusted for BMI, smoking status, and alcohol consumption; Model 3 further adjusted for existing comorbidities including hypertension, dyslipidemia, diabetes, and CKD. To address potential multicollinearity concerns in Model 3, generalized variance inflation factors (GVIF) were calculated for all covariates, with GVIF^(1/(2 × DF)) < 2.5 considered acceptable. Inter-disease correlations and comorbidity patterns were also assessed to evaluate disease independence in the study population.

Individual SDOH component analyses were performed to explore the relationship between each SDOH domain and CKM stages. Separate multinomial logistic regression analyses were conducted for educational attainment, marital status, employment status, household income, and community type, each using the same three-model adjustment strategy described above.

Subgroup analyses were stratified by age, sex, smoking status, and alcohol consumption, and age-adjusted models were additionally estimated. Sensitivity analyses were conducted using the original dataset before imputation to evaluate the robustness of findings. The same three multinomial logistic regression models were applied to the complete-case data to determine whether imputation procedures influenced the observed associations between SDOH and CKM stages.

Statistical significance was set at *p* < 0.05. All analyses were performed using *R* software (version 4.3.0).

## Results

### Participant characteristics

A total of 4,066 participants were included from the STONE population, with a mean age of 41.7 (12.1) years, among whom 2,291 (56.3%) were female and 1,775 (43.7%) were male. The distribution across CKM stages was: CKM-0, 843 (20.7%); CKM-1, 487 (12.0%); CKM-2, 1814 (44.6%); and CKM-3/4, 922 (22.7%) (details in Table 1). Senior male citizens with higher BMI are more likely in advanced CKM stages (all *p* < 0.001). Lifestyle risk factors increased with advancing CKM stages: smoking prevalence rose from 11.5% in CKM-0 to 25.9% in CKM-3/4, and alcohol consumption increased from 14.7 to 26.8% (both *p* < 0.001).

**Table 1:** Characteristics of participants according to CKM stages.

Characteristics	Total (*n* = 4,066)	CKM-0 (*n* = 843)	CKM-1 (*n* = 487)	CKM-2 (*n* = 1814)	CKM-3/4 (*n* = 922)	*p* value
Age (years), M ± SD	41.7 ± 12.1	33.9 ± 9.3	38.6 ± 11.2	40.2 ± 10.9	53.6 ± 7.8	< 0.001
Male, *n* (%)	1775 (43.7)	244 (28.9)	147 (30.2)	916 (50.5)	468 (50.8)	< 0.001
Education, *n* (%)						< 0.001
Lower	3,123 (76.8)	777 (92.2)	390 (80.1)	1,412 (77.8)	544 (59)	
Higher	943 (23.2)	66 (7.8)	97 (19.9)	402 (22.2)	378 (41)	
Marriage, *n* (%)						< 0.001
Coupled	3,349 (82.4)	580 (68.8)	389 (79.9)	1,516 (83.6)	864 (93.7)	
Single	717 (17.6)	263 (31.2)	98 (20.1)	298 (16.4)	58 (6.3)	
Employment status, *n* (%)						< 0.001
Employed or student	3,115 (76.6)	776 (92.1)	390 (80.1)	1,484 (81.8)	465 (50.4)	
Unemployed or retired	951 (23.4)	67 (7.9)	97 (19.9)	330 (18.2)	457 (49.6)	
Household income, *n* (%)						< 0.001
Higher	3,135 (77.1)	700 (83)	377 (77.4)	1,425 (78.6)	633 (68.7)	
Lower	931 (22.9)	143 (17)	110 (22.6)	389 (21.4)	289 (31.3)	
Community, *n* (%)						0.184
Urban	1,416 (34.8)	268 (31.8)	170 (34.9)	641 (35.3)	337 (36.6)	
Rural or suburban	2,650 (65.2)	575 (68.2)	317 (65.1)	1,173 (64.7)	585 (63.4)	
Unfavorable SDOH, *n* (%)						< 0.001
≤ 2	3,326 (81.8)	759 (90)	410 (84.2)	1,533 (84.5)	624 (67.7)	
> 2	740 (18.2)	84 (10)	77 (15.8)	281 (15.5)	298 (32.3)	
Smoking status, *n* (%)						< 0.001
Never	3,124 (76.8)	731 (86.7)	420 (86.2)	1,364 (75.2)	609 (66.1)	
Quit	170 (4.2)	15 (1.8)	14 (2.9)	67 (3.7)	74 (8)	
Current	772 (19.0)	97 (11.5)	53 (10.9)	383 (21.1)	239 (25.9)	
Alcohol consumption, *n* (%)						< 0.001
No	3,177 (78.1)	719 (85.3)	411 (84.4)	1,372 (75.6)	675 (73.2)	
Yes	889 (21.9)	124 (14.7)	76 (15.6)	442 (24.4)	247 (26.8)	
BMI (kg/m2), M ± SD	24.2 ± 10.5	21.0 ± 1.9	25.3 ± 3.6	24.7 ± 6.5	25.6 ± 19.6	< 0.001
Diabetes, *n* (%)	328 (8.1)	1 (0.1)	22 (4.5)	147 (8.1)	158 (17.1)	< 0.001
Dyslipidemia, *n* (%)	994 (24.4)	62 (7.4)	58 (11.9)	551 (30.4)	323 (35)	< 0.001
Hypertension, *n* (%)	822 (20.2)	0 (0)	0 (0)	541 (29.8)	281 (30.5)	< 0.001
CKD, *n* (%)	270 (6.6)	0 (0)	0 (0)	191 (10.5)	79 (8.6)	< 0.001

Regarding SDOH distribution, the percentage of individuals with unfavorable overall SDOH status (score ≥2) increased from 10.0% in CKM-0 to 32.3% in CKM-3/4 (*p* < 0.001). Individual SDOH components showed distinct patterns: lower educational attainment and unemployment increased with advancing CKM stages, while the proportion of subjects unmarried or living alone decreased, reflecting the older age profile of advanced stages. Community type showed no significant difference across CKM stages (*p* = 0.184).

**Figure 2:**
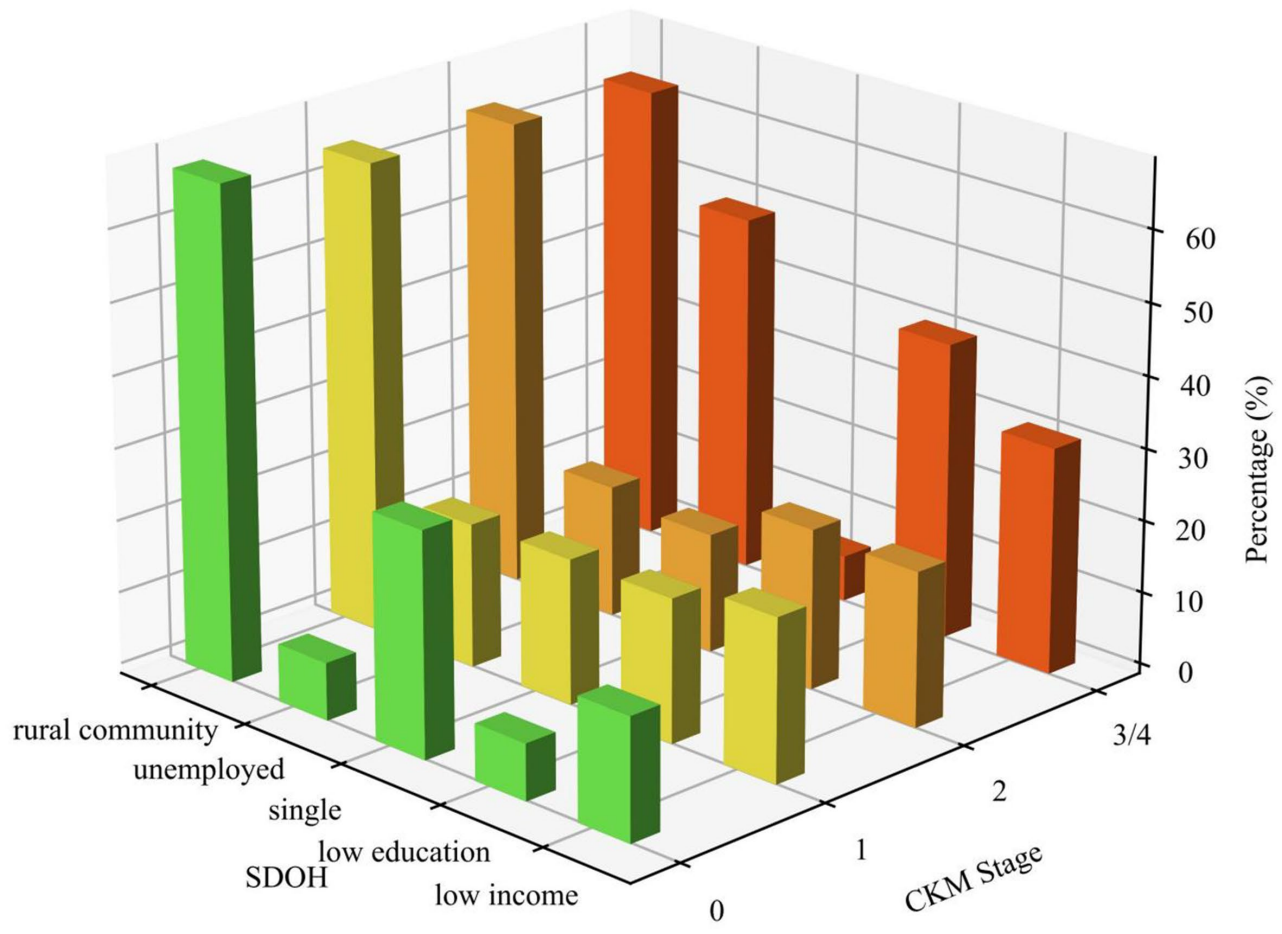
Barplot of unfavorable SDOH composition in CKM-0-4.

The barplot (Figure 2) illustrates the distribution of SDOH factors across different CKM stages, showing that initial CKM stages were more prevalent among individuals with higher education and income levels, while later stages showed increased proportions of participants from rural/suburban communities and among unmarried individuals.

### Associations between SDOH and CKM staging

**Figure 3:**
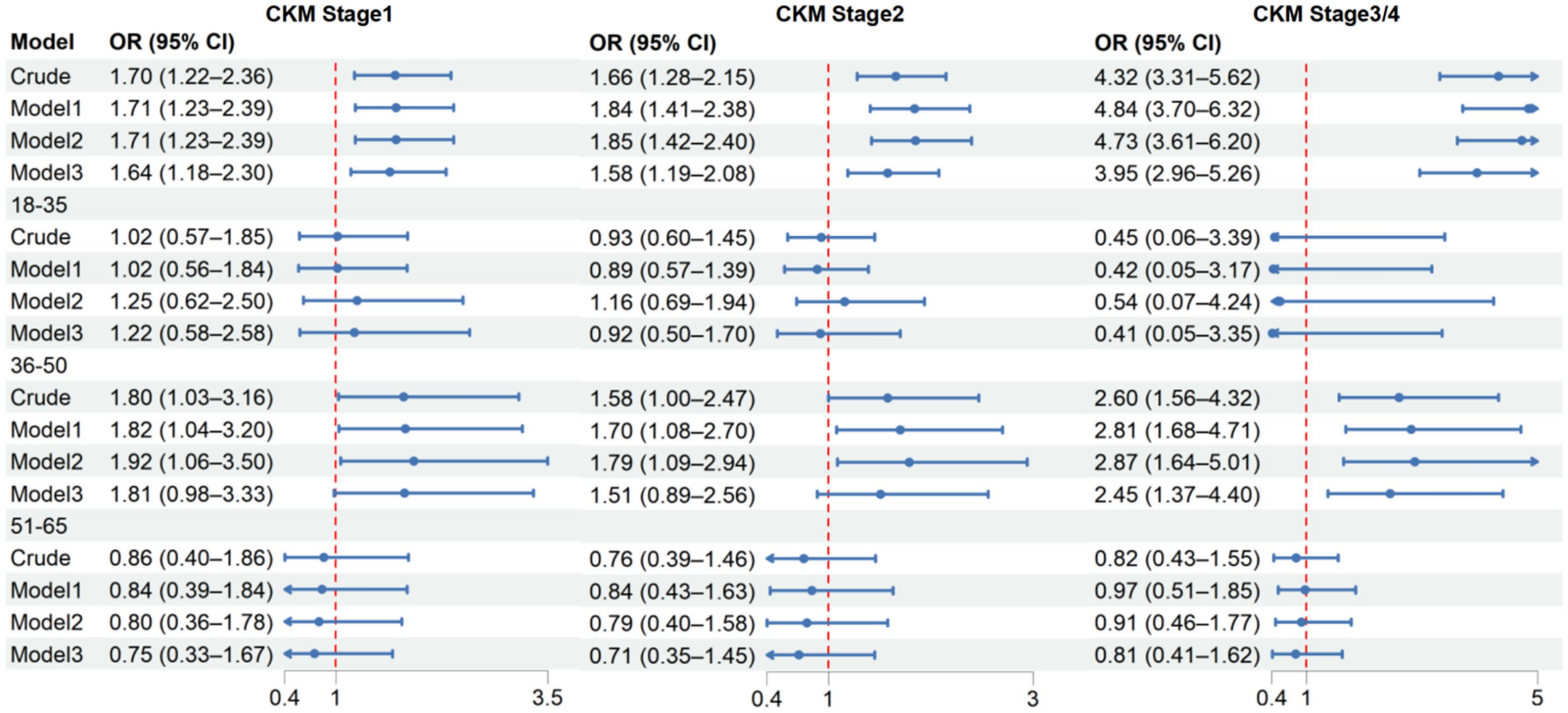
Age-stratified associations between SDOH and CKM syndrome stages. Model 1: gender Model 2: gender, BMI, smoking status, alcohol consumption Model 3: gender, BMI, smoking status, alcohol consumption, hypertension, dyslipidemia, diabetes, CKD.

Multinomial logistic regression analysis revealed significant associations between unfavorable SDOH and CKM staging (Figure 3). In the crude model, individuals with unfavorable Compared to CKM-0, SDOH had increased odds of 1.70 (95% confidence interval (CI): 1.22–2.36) for CKM CKM-1, 1.66 (95% CI: 1.28–2.15) for CKM-2, and 4.32 (95% CI: 3.31–5.62) for CKM-3/4. After full adjustment for gender, BMI, smoking status, alcohol consumption, and chronic metabolic disorders in Model 3, the associations remained significant with odd ratios (ORs) of 1.64 (95% CI: 1.18–2.30), 1.58 (95% CI: 1.19–2.08), and 3.95 (95% CI: 2.96–5.26), respectively.

Regarding individual SDOH components, crude analyses showed the strongest associations with advanced CKM stages (CKM-3/4) for educational attainment and employment status (Table 2). Lower educational attainment was associated with an 8.18-fold increased odds (95% CI: 6.16–10.86), while unemployment/retirement showed an 11.38-fold increased odds (95% CI: 8.59–15.08). Conversely, being married or living with a partner was protective (OR: 0.15, 95% CI: 0.11–0.20). Lower household income approximately doubled the risk (OR: 2.24, 95% CI: 1.78–2.81), while rural/suburban residence showed a modest protective association (OR: 0.81, 95% CI: 0.66–0.99). Multivariable-adjusted analyses for individual SDOH components (Supplementary Table 1) demonstrated consistent trends with attenuated but significant associations across all models.

**Table 2:** Associations between five unfavorable SDOH and CKM Syndrome stages.

Unfavorable SDOH	CKM syndrome stage			
	Stage0	Stage1	Stage2	Stage3/4
Educational attainment	1.00 (reference)	2.93 (2.09–4.1)	3.35 (2.55–4.41)	8.18 (6.16–10.86)
Marital status	1.00 (reference)	0.56 (0.43–0.72)	0.43 (0.36–0.52)	0.15 (0.11–0.2)
Employment status	1.00 (reference)	2.88 (2.06–4.02)	2.58 (1.95–3.4)	11.38 (8.59–15.08)
Household income	1.00 (reference)	1.43 (1.08–1.89)	1.34 (1.08–1.65)	2.24 (1.78–2.81)
Community type	1.00 (reference)	0.87 (0.69–1.1)	0.85 (0.72–1.02)	0.81 (0.66–0.99)

VIF analysis confirmed the absence of problematic multicollinearity among Model 3 variables, with all GVIF^(1/(2 × DF)) values well below 2.5 (Supplementary Table 3). Inter-disease correlations were weak (r < 0.15 for all pairs, Supplementary Figure 1), and comorbidity analysis revealed low co-occurrence rates, with 71.2% of participants having no chronic diseases (Supplementary Figure 2).

### Age-specific analysis

Further stratified analysis by age groups confirmed that SDOH associations with CKM staging were confined to middle-aged adults, with no significant associations observed in younger or older groups (Figure 3). In the middle-aged group, unfavorable SDOH remained significantly associated with advanced CKM stages even after full adjustment (OR = 2.45, 95% CI: 1.37–4.40), suggesting age-dependent effects of social determinants on metabolic health.

### Subgroup analysis

**Figure 4:**
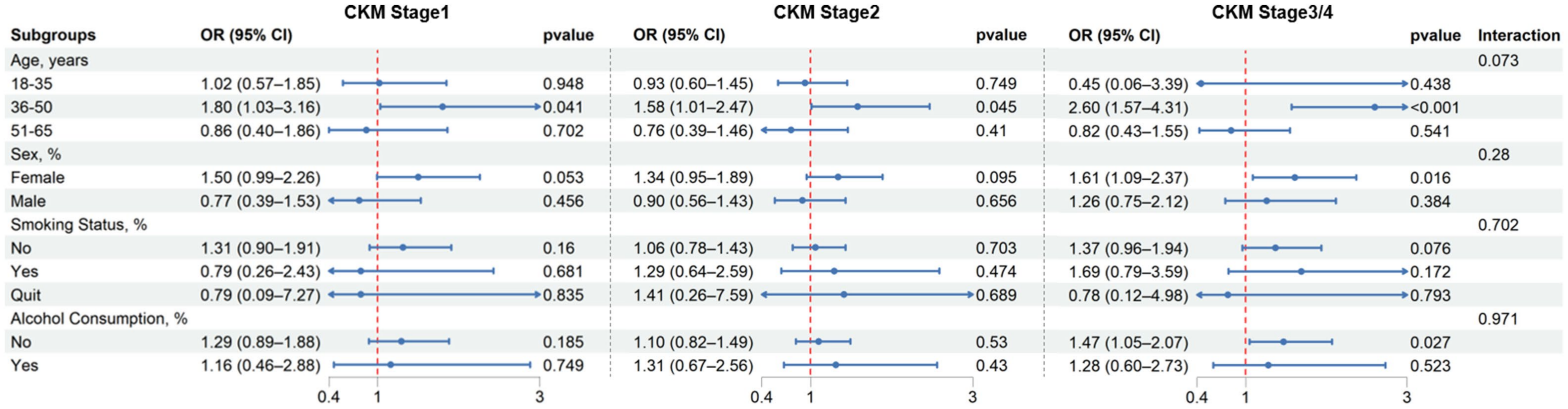
Subgroup analysis of associations between unfavorable SDOH and CKM syndrome stages by age, sex, smoking, and drinking status.

Subgroup analyses revealed heterogeneous associations between unfavorable SDOH and CKM staging across different population subgroups (Figure 4). The associations were present only among individuals aged 35–51 years, with significant ORs across all CKM stages (CKM-1: OR = 1.80, *p* = 0.041; CKM-2: OR = 1.58, *p* = 0.045; CKM-3/4: OR = 2.60, *p* < 0.001). No significant associations were observed in < 35 years old or ≥ 50 groups.

Sex-stratified results were consistent with the main findings: women showed stronger associations—most evident in advanced CKM stages (OR = 1.61, *p* = 0.016)—while associations in men were weaker and nonsignificant across stages. Likewise, unfavorable SDOH were more evident among non-smokers and non-drinkers, whereas current smokers and drinkers showed associations mainly in advanced stages. These patterns remained largely unchanged after age adjustment. No statistically significant interactions were detected (all *P* for interaction > 0.05).

### Sensitivity analysis

Sensitivity analysis using the original pre-imputation dataset with missing data handled as dummy variables yielded consistent results (Supplementary Table 2). In the fully adjusted model, unfavorable SDOH remained significantly associated with higher CKM stages, with ORs of 1.57 (95% CI: 1.07–2.30), 1.78 (95% CI: 1.26–2.52), and 4.33 (95% CI: 3.07–6.12) for stages 1, 2, and 3–4, respectively. These findings confirm the robustness of the primary analysis results.

## Discussion

This epidemiological study based on Shanghai community population revealed that individuals with unfavorable SDOH, especially low education level, unemployment, and low income, exhibited higher risks of CKM progression compared with those with favorable SDOH. These associations were present only in the 36–50 age group. Among individuals free from lifestyle risk factor (non-drinkers) and women individuals, significant associations was demonstrated between unfavorable SDOH and advanced CKM stages, whereas men showed no associations across all CKM stages. This suggests that in the absence of dominant lifestyle risk factors, social determinants exert earlier metabolic effects in women compared to men.

Previous studies have shown links between adverse SDOH and the prevalence and risk of major components of CKM syndrome (21–26). Further studies have connected social disadvantages with a wider spectrum of risk factors for CVD and the occurrence of CVD events (14, 27). Additionally, negative correlations between SDOH and CKD have been identified (28, 29). It is noteworthy that in the presidential advisory, the AHA underscored that adverse SDOH can precipitate downstream cardiovascular events and cardiovascular related mortality, while early intervention targeting SDOH is expected to release the CKM burden by improving health behavior and access to healthcare (4). Therefore, utilizing community population, this study conducted a comprehensive evaluation of associations between multi-dimensional SDOH and CKM stages in China for the first time. While characterized by the clustering in the initial phases of CKM, the research based on community sample is able to detect the underlying effects of SDOH on CKM at an earlier stage, offering precise evidence for primary preventive measures.

The study demonstrates the association between low education level, retired or unemployment, and low income—and CKM higher stages, which aligns with global trends. People with higher educational attainment typically display greater health literacy and more proactive self-care, and they usually command higher earnings. By contrast, restricted income can compromise food security, healthcare access, and even educational opportunities, thereby influencing CKM health. Unemployment or retirement tightens financial margins. Furthermore, sustained exposure can incubate anxiety, depression, and other psychological strains that further undermine cardiovascular health (25). Interventions must therefore be holistic, improving education attainment, expanding employment prospects, and elevating incomes simultaneously, rather than targeting any single factor in isolation. Whereas, unlike some western reports, we observed that the individuals who were unmarried, divorced, or widowed exhibited a lower CKM risk in Chinese population. This discrepancy likely reflects cultural norms and distinct social support fabrics, such as the strong family support system in China that may buffer the CKM risk associated with being unmarried.

Age-stratified analysis indicates that the influence of SDOH is contingent upon age, with individuals in the middle age of 36–50 showing the highest sensitivity to SDOH exposure, which could be attributed to the concurrent pressures of career demands, family responsibilities, and shifts in societal roles that are characteristic of this demographic. The diminished link between SDOH and CKM staging in younger and older age groups implies that distinct metabolic risk factors may predominate at different life stages, with biological aging playing a more major role in the older population, and therefore suggest that targeted SDOH interventions could be instrumental in mitigating CKM advancement for specific age gaps.

Lifestyle acts as a pivotal mediator between SDOH and cardiovascular health (30, 31). It is crucial to emphasize that smoking and alcohol consumption remain well-established harmful factors for cardiovascular and metabolic health (32, 33). Our subgroup findings suggest that among individuals without these lifestyle risk behaviors, social determinants demonstrate differential effects by sex. In women with healthier lifestyles, socioeconomic disadvantages (low education, unemployment, limited income) translate into elevated risk of CKM stage3/4, whereas in men, social determinants show no significant effects in the association with CKM. These insights provide a clear rationale for precision prevention: public health efforts should move beyond generic health education to implement targeted interventions. Policies addressing education attainment, employment opportunities, and income security—particularly for women and middle-aged adults—could serve as potent levers for CKM health management, especially among populations without concurrent lifestyle risk factors.

Nevertheless, the cross-sectional design precludes establishing temporal relationships and leaves the study susceptible to reverse causality. For example, people who experience incipient metabolic disturbances may be more health-conscious and, consequently, more likely to adopt or maintain salutary behaviors such as abstaining from tobacco and alcohol. Additionally, the assessment of SDOH was incomplete concerning data on physical activity, food security, and neighborhood violence were not collected at baseline; omission of these unmeasured social domains may leave uncontrolled confounding bias and restrict generalizability to populations where such factors meaningfully shape cardiometabolic risk. Future research will require longitudinal cohort designs to gain a deeper understanding of the biological pathways through which social environmental factors impact metabolic health.

## Conclusion

Unfavorable SDOH, especially low educational attainment, unemployment or retirement, and limited income, robustly accelerate the advancement of CKM syndrome. These relationships are modulated by age and sex: adults between 36 and 50 years are most vulnerable. Among women and non-drinker, elevated risk appears in advanced CKM stage, whereas men did not showed this pattern, suggesting sex-specific metabolic vulnerabilities to social determinants.

## Data Availability

The original contributions presented in the study are included in the article/Supplementary material, further inquiries can be directed to the corresponding authors.

## Footnotes

1
https://tjj.sh.gov.cn/
