# Supplementary material for: Low education and employment status drive cardiometabolic health: a social determinants perspective in Chinese population
Source: Front Public Health. 2025 Dec 10;13:1676833. doi: 10.3389/fpubh.2025.1676833 (PMC12727962; doi:10.3389/fpubh.2025.1676833)
Supplement: Supplementary file 1 [file Supplementary_file_1.docx]

**Legends**

**Supplementary Table 1.** Adjusted associations between individual SDOH components and CKM syndrome stages.

**Supplementary Table 2.** Sensitivity Analysis - Associations between unfavorable SDOH and CKM syndrome stages.

**Supplementary Table 3.** Variance Inflation Factor Analysis for Model 3 Variables.

**Supplementary Figure 1.** Correlation Matrix of Comorbid Conditions in the Study Population.

**Supplementary Figure 2.** Distribution of Comorbidity Patterns Among Study Participants.

**Supplementary Table 1.** Adjusted associations between individual SDOH components and CKM syndrome stages.

| **Items** | **CKM syndrome stage** | | | |
| --- | --- | --- | --- | --- |
|  | **Stage 0** | **Stage 1** | **Stage 2** | **Stage 3/4** |
| Educational attainment | 1.00 (reference) | 2.93 (2.09-4.1) | 3.35 (2.55- 4.41) | 8.18 (6.16- 10.86) |
| Marital status | 1.00 (reference) | 0.56 (0.43-0.72) | 0.43 (0.36- 0.52) | 0.15 (0.11- 0.2) |
| Employment status | 1.00 (reference) | 2.88 (2.06-4.02) | 2.58 (1.95- 3.4) | 11.38 (8.59- 15.08) |
| Household income | 1.00 (reference) | 1.43 (1.08-1.89) | 1.34 (1.08- 1.65) | 2.24 (1.78- 2.81) |
| Community type | 1.00 (reference) | 0.87 (0.69-1.1) | 0.85 (0.72- 1.02) | 0.81 (0.66- 0.99) |
| **Model 1** |  |  |  |  |
| Educational attainment | 1.00 (reference) | 2.94 (2.1-4.11) | 3.48 (2.64- 4.59) | 8.51 (6.4- 11.33) |
| Marital status | 1.00 (reference) | 0.55 (0.42-0.72) | 0.41 (0.34- 0.5) | 0.14 (0.1- 0.19) |
| Employment status | 1.00 (reference) | 2.97 (2.12-4.17) | 3.25 (2.45- 4.3) | 15.81 (11.81- 21.16) |
| Household income | 1.00 (reference) | 1.43 (1.08-1.89) | 1.38 (1.11- 1.7) | 2.3 (1.83- 2.9) |
| Community type | 1.00 (reference) | 0.87 (0.68-1.1) | 0.81 (0.68- 0.97) | 0.77 (0.63- 0.94) |
| **Model 2** |  |  |  |  |
| Educational attainment | 1.00 (reference) | 2.24 (1.57-3.19) | 2.67 (1.99- 3.59) | 6.36 (4.68- 8.63) |
| Marital status | 1.00 (reference) | 0.69 (0.51-0.92) | 0.52 (0.42- 0.65) | 0.18 (0.13- 0.25) |
| Employment status | 1.00 (reference) | 2.45 (1.72-3.5) | 2.71 (2.01- 3.66) | 12.77 (9.35- 17.44) |
| Household income | 1.00 (reference) | 1.42 (1.05-1.92) | 1.39 (1.09- 1.76) | 2.28 (1.77- 2.95) |
| Community type | 1.00 (reference) | 1.10 (0.85-1.42) | 1.01 (0.83- 1.23) | 0.94 (0.75- 1.17) |
| **Model 3** |  |  |  |  |
| Educational attainment | 1.00 (reference) | 2.19 (1.53-3.14) | 2.19 (1.6- 2.99) | 5.03 (3.64- 6.95) |
| Marital status | 1.00 (reference) | 0.61 (0.46-0.82) | 0.49 (0.38- 0.63) | 0.18 (0.13- 0.25) |
| Employment status | 1.00 (reference) | 2.42 (1.68-3.47) | 2.32 (1.69- 3.18) | 10.97 (7.91- 15.21) |
| Household income | 1.00 (reference) | 1.41 (1.04-1.91) | 1.26 (0.97- 1.63) | 2.02 (1.54- 2.67) |
| Community type | 1.00 (reference) | 1.10 (0.85-1.43) | 0.89 (0.72- 1.1) | 0.80 (0.63- 1.02) |
| ^a^Model 1: Gender  ^b^Model 2: Gender, BMI, smoking status, alcohol consumption  ^c^Model 3: Gender, BMI, smoking status, alcohol consumption, hypertension, dyslipidemia, diabetes, CKD. | | | | |

**Supplementary Table 2.** Sensitivity Analysis - Associations between unfavorable SDOH and CKM syndrome stages.

|  | **CKM syndrome stage** | | | |
| --- | --- | --- | --- | --- |
|  | **Stage 0** | **Stage 1** | **Stage 2** | **Stage 3/4** |
| **Crude** | 1.00 (reference) | 1.7 (1.22 ~ 2.36) | 1.77 (1.36 ~ 2.31) | 4.32 (3.31 ~ 5.62) |
| **Model 1^a^** | 1.00 (reference) | 1.71 (1.23 ~ 2.39) | 2.04 (1.55 ~ 2.67) | 4.91 (3.75 ~ 6.43) |
| **Model 2^b^** | 1.00 (reference) | 1.67 (1.16 ~ 2.40) | 2.02 (1.49 ~ 2.74) | 4.68 (3.45 ~ 6.35) |
| **Model 3^c^** | 1.00 (reference) | 1.57 (1.07 ~ 2.30) | 1.78 (1.26 ~ 2.52) | 4.33 (3.07 ~ 6.12) |
| ^a^Model 1: Gender  ^b^Model 2: Gender, BMI, smoking status, alcohol consumption  ^c^Model 3: Gender, BMI, smoking status, alcohol consumption, hypertension, dyslipidemia, diabetes, CKD. | | | | |

**Supplementary Table 3.** Variance Inflation Factor Analysis for Model 3 Variables.

| **Items** | **GVIF** | **DF** | **GVIF^(1/(2×DF))** | **Coefficient Change (%)*** |
| --- | --- | --- | --- | --- |
| **SDOH** | | | | |
| Sex | 1.587 | 1 | 1.260 | -15.4 |
| BMI | 1.014 | 1 | 1.007 | -2.5 |
| Alcohol consumption | 1.277 | 1 | 1.130 | -0.2 |
| Smoking history | 1.536 | 2 | 1.113 | 0.3 |
| Hypertension | 1.076 | 1 | 1.038 | 10.2 |
| Diabetes mellitus | 1.069 | 1 | 1.034 | 5.2 |
| Chronic kidney disease | 1.030 | 1 | 1.015 | 10.6 |
| Cardiovascular disease | 1.105 | 1 | 1.051 | 299.7 |
| **Educational attainment** | | | | |
| Sex | 1.582 | 1 | 1.258 | -6.1 |
| BMI | 1.016 | 1 | 1.008 | 3.3 |
| Alcohol consumption | 1.277 | 1 | 1.13 | 0 |
| Smoking history | 1.545 | 2 | 1.115 | 0.4 |
| Hypertension | 1.078 | 1 | 1.038 | 12.3 |
| Diabetes mellitus | 1.074 | 1 | 1.037 | 4.2 |
| Chronic kidney disease | 1.031 | 1 | 1.016 | 7.6 |
| Cardiovascular disease | 1.111 | 1 | 1.054 | 166.5 |
| **Marital status** | | | | |
| Sex | 1.583 | 1 | 1.258 | -4.6 |
| BMI | 1.015 | 1 | 1.007 | 1.9 |
| Alcohol consumption | 1.279 | 1 | 1.131 | -0.1 |
| Smoking history | 1.536 | 2 | 1.113 | 0.1 |
| Hypertension | 1.077 | 1 | 1.038 | 6 |
| Diabetes mellitus | 1.068 | 1 | 1.034 | 1.4 |
| Chronic kidney disease | 1.028 | 1 | 1.014 | -0.1 |
| Cardiovascular disease | 1.089 | 1 | 1.044 | 86.6 |
| **Employment status** | | | | |
| Sex | 1.627 | 1 | 1.275 | -17.9 |
| BMI | 1.014 | 1 | 1.007 | 0.4 |
| Alcohol consumption | 1.277 | 1 | 1.13 | 0 |
| Smoking history | 1.536 | 2 | 1.113 | 0 |
| Hypertension | 1.077 | 1 | 1.038 | 8.1 |
| Diabetes mellitus | 1.067 | 1 | 1.033 | 1.1 |
| Chronic kidney disease | 1.029 | 1 | 1.014 | 3 |
| Cardiovascular disease | 1.198 | 1 | 1.095 | 232.9 |
| **Household income** | | | | |
| Sex | 1.579 | 1 | 1.256 | -13 |
| BMI | 1.014 | 1 | 1.007 | -0.1 |
| Alcohol consumption | 1.277 | 1 | 1.13 | -0.1 |
| Smoking history | 1.534 | 2 | 1.113 | 0.2 |
| Hypertension | 1.078 | 1 | 1.038 | 44.8 |
| Diabetes mellitus | 1.068 | 1 | 1.033 | 8.2 |
| Chronic kidney disease | 1.028 | 1 | 1.014 | 7.4 |
| Cardiovascular disease | 1.077 | 1 | 1.038 | 329.5 |
| **Community type** | | | | |
| Sex | 1.584 | 1 | 1.258 | 1 |
| BMI | 1.276 | 1 | 1.13 | 0 |
| Alcohol consumption | 1.542 | 2 | 1.114 | -0.9 |
| Smoking history | 1.033 | 1 | 1.016 | -3.9 |
| Hypertension | 1.079 | 1 | 1.039 | -2.9 |
| Diabetes mellitus | 1.084 | 1 | 1.041 | 8.8 |
| Chronic kidney disease | 1.081 | 1 | 1.04 | -20.5 |
| Cardiovascular disease | 1.075 | 1 | 1.037 | 69.3 |

Note: GVIF (Generalized Variance Inflation Factor) and GVIF^(1/(2×DF)) are dimensionless diagnostic statistics for assessing multicollinearity. Coefficient Change (%) represents the percentage change in regression coefficients when each variable is removed from the model.


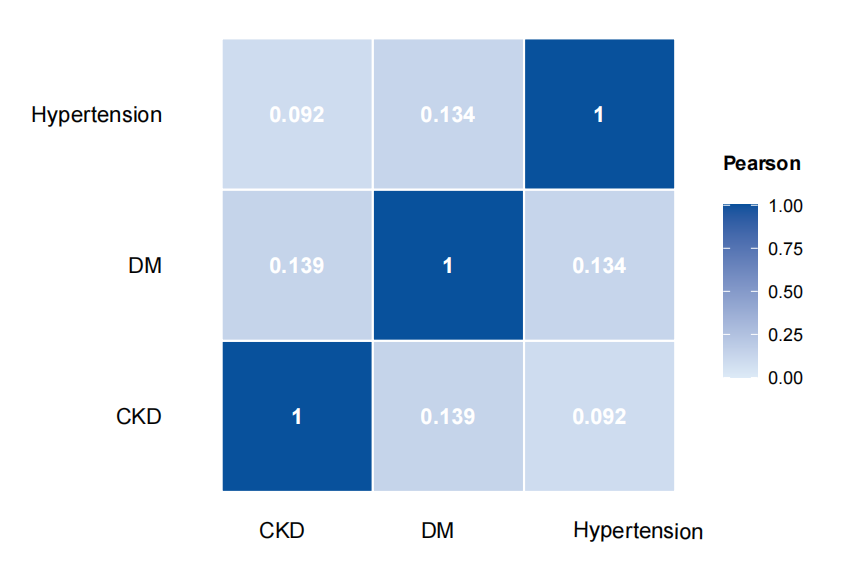


**Supplementary Figure 1.** Correlation Matrix of Comorbid Conditions in the Study Population.


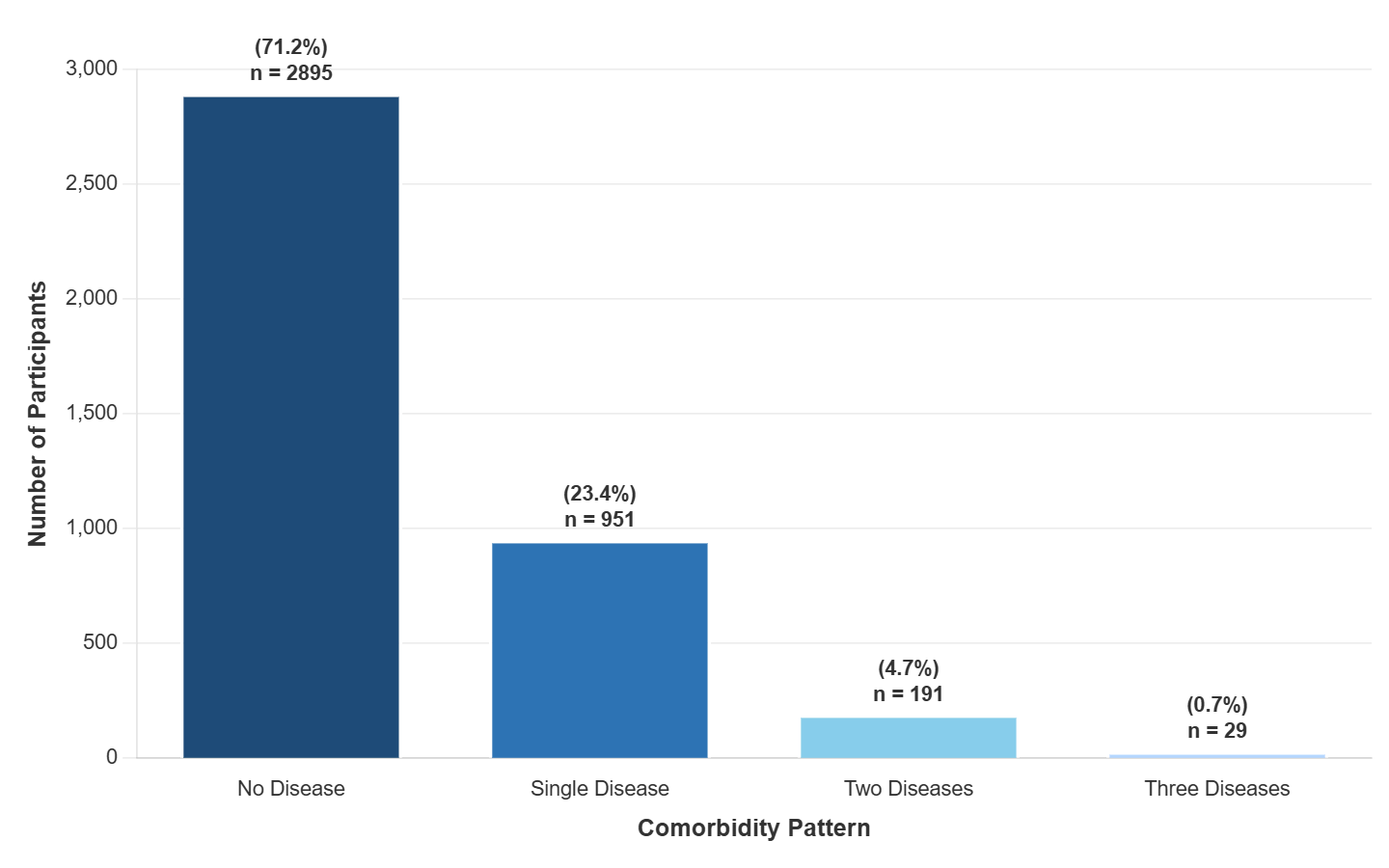
**Supplementary Figure 2.** Distribution of Comorbidity Patterns Among Study Participants.
